# Calcineurin Antagonizes AMPK to Regulate Lipolysis in *Caenorhabditis elegans*

**DOI:** 10.3390/molecules22071062

**Published:** 2017-06-26

**Authors:** Yanli Wang, Cangsang Xie, Zhiqing Diao, Bin Liang

**Affiliations:** 1 Key Laboratory of Animal Models and Human Disease Mechanisms of the Chinese Academy of Sciences & Yunnan Province, Kunming Institute of Zoology, Chinese Academy of Sciences, Kunming 650223, China; 035wangyanli@163.com (Y.W.); ywowen@126.com (C.X.); datangchenjian@163.com (Z.D.); 2 Kunming College of Life Science, University of Chinese Academy of Sciences, Kunming 650204, China; 3 School of Life Sciences, Anhui University, Hefei 230601, China

**Keywords:** calcineurin, tacrolimus, AMP-activated protein kinase (AMPK), triglyceride lipase ATGL, lipolysis

## Abstract

Calcineurin is a calcium- and calmodulin-dependent serine/threonine protein phosphatase, and the target of immunosuppressive agent tacrolimus (TAC). The dysfunction of calcineurin, or clinical applications of tacrolimus, have been reported to be associated with dyslipidemia. The underlying mechanisms of calcineurin and tacrolimus in lipid metabolism are largely unknown. Here, we showed that mutations of *tax-6* and *cnb-1*, which respectively encode the catalytic subunit and the regulatory subunit of calcineurin, together with tacrolimus treatment, consistently led to decreased fat accumulation and delayed growth in the nematode *Caenorhabditis elegans*. In contrast, disruption of the AMP-activated protein kinase (AMPK) encoded by *aak-1* and *aak-2* reversed the above effects in worms. Moreover, calcineurin deficiency and tacrolimus treatment consistently activated the transcriptional expression of the lipolytic gene *atgl-1*, encoding triglyceride lipase. Furthermore, RNAi knockdown of *atgl-1* recovered the decreased fat accumulation in both calcineurin deficient and tacrolimus treated worms. Collectively, our results reveal that immunosuppressive agent tacrolimus and their target calcineurin may antagonize AMPK to regulate ATGL and lipolysis, thereby providing potential therapy for the application of immunosuppressive agents.

## 1. Introduction

The Ca^2+^/calmodulin-dependent phosphatase calcineurin is a heterodimer that consists of a catalytic calmodulin-binding subunit calcineurin A (CnA) and a regulatory Ca^2+^-binding subunit, calcineurin B (CnB) [1,2]. As a phosphatase, calcineurin acts with many proteins involved in Ca^2+^-dependent signal transduction, as well as numerous cellular processes and physiologies. Dysfunction of calcineurin has been reported to be associated with a number of diseases, such as cardiac and kidney hypertrophy [3,4], Alzheimer’s disease [5], schizophrenia [6], Duchenne muscular dystrophy [7], and diabetes [4,8]. In addition, deletion of *CNA1* leads to reduced levels of lipids with short-chain fatty acids in yeast [9]. Similarly, mice lacking calcineurin Aβ *CnA*β*(^−/−^)* display consistent hyperlipidemia with elevated levels of plasma triglyceride, cholesterol, and free fatty acids [10].

Calcineurin is the target of the immunosuppressant drug tacrolimus (TAC) [11], which has been widely used for organ transplantation because of its ability to prevent immune responses. Tacrolimus (TAC) binds and inhibits calcineurin, thereby interfering with T-cell responses to antigen [12,13,14]. However, clinical applications of immunosuppressant drug tacrolimus (TAC) have been reported to be accompanied with unexpected side effects, especially the development of metabolic complications, such as dyslipidemia [15,16]. Tacrolimus (TAC) has also been associated with lower LDL-c, apolipoprotein B, and triglyceride levels [17,18]. Taken together, these studies have coherently demonstrated that the immunosuppressant drug tacrolimus (TAC), as well as its target calcineurin, apparently participate in the process of regulating lipid metabolism, whereas the underlying mechanisms still remains largely unknown.

The sequences and functions of calcineurin are highly conserved from yeast to human [2]. The nematode, *Caenorhabditis elegans* contains *tax-6* and *cnb-1* genes that encode the catalytic subunit and the regulatory subunit with high amino acid identities to respective human calcineurin A and calcineurin B [19,20,21]. Similar to its mammalian homologues, *C. elegans* calcineurin also plays pivotal roles in regulating a variety of cellular processes involving in development, fertility, proliferation, behaviors, and lifespan [20,22]. However, whether or not TAX-6 or CNB-1 is also involved in lipid metabolism has not been characterized.

In this study, we took the advantages of *C. elegans* as a genetically-tractable model to investigate the roles of calcineurin as well as its inhibitor tacrolimus (TAC) in lipid metabolism. We showed that either calcineurin-deficient mutants or tacrolimus (TAC)-treated worms displayed consistent reduction of fat accumulation. Furthermore, we found that the inactivation of AMP-activated protein kinase AMPK or its target adipose triglyceride lipase ATGL-1 reverses the fat-lowering effect of calcineurin and its inhibitors.

## 2. Results

### 2.1. Genetic Disruption of Calcineurin Reduces Fat Accumulation in C. elegans

**Figure 1:**
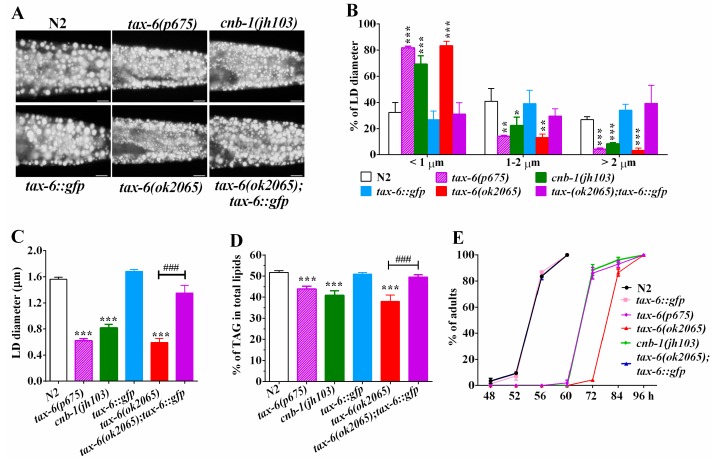
Loss-of-function of calcineurin genes *tax-6* or *cnb-1* exhibited a remarkable reduction in fat accumulation. (**A**) Nile Red staining of fixed worms. Representative animals with stained lipid droplets (LDs) in the posterior region. In all of the represented animals, the anterior is indicated on the left and the posterior is indicated on the right. Scale bar represents 10 μm; (**B**) Distribution of the lipid droplets (% lipid droplets) were measured from Nile Red staining of fixed worms from (**A**), *n* = 10 for each worm strain; (**C**) The average size of the lipid droplets (LD) were measured from Nile Red staining of fixed worms from (**A**), *n* = 10 for each worm strain; (**D**) percentage of triacylglycerol (TAG) in total lipids (TAG + phospholipids). Data are presented as the means ± SD of at least three biological repeats; and (**E**) The growth rate of N2, *tax-6(ok2065)*, *cnb-1(jh103)* and *tax-6(p675)* worms. *n* > 150 worms. Data are presented as the means ± SD. * indicates significant difference between wild-type N2 and a specific worm strain, * *p* < 0.05, ** *p* < 0.01, *** *p* < 0.001. ^#^ indicates significant difference between two indicated worm strains, ^###^ *p* < 0.001.

To examine whether calcineurin plays a role in regulating lipid metabolism in *C. elegnas*, we investigated the fat storage of calcineurin deficient mutants with the established methodologies of Nile Red staining of fixed worms, as well as thin-layer chromatography and gas chromatography (TLC/GC) [23,24,25,26]. In *C. elegans*, *tax-6* and *cnb-1* genes encode the catalytic subunit and the regulatory subunit of calcineurin, respectively [19,20,21]. Nile Red staining of fixed worms showed that all three calcineurin deficient mutants *tax-6(ok2065)*, *tax-6(p675)*, and *cnb-1(jh103)* displayed apparently reduced fat accumulation compared to that of the wild-type N2 (Figure 1A). Quantification of lipid droplets (LDs) further revealed that *tax-6(ok2065)*, *tax-6(p675)* and *cnb-1(jh103)* mutant worms displayed a dramatic increase in the percentage of smaller size LDs (<1 μm), but decreased percentage of middle (1–2 μm) and larger LDs (>2 μm) (Figure 1B), leading to significantly reduced LD size (Figure 1C). The average LD size was 1.56 ± 0.03 μm in N2, while those in *tax-6(ok2065)*, *tax-6(p675)*, and *cnb-1(jh103)* mutants were 0.60 ± 0.05, 0.62 ± 0.05, and 0.82 ± 0.08 μm, respectively (Figure 1C). Likewise, lipid analysis by TLC/GC further confirmed that the levels of triacylglyceride (TAG) significantly decreased in *tax-6(ok2065)* (37.9 ± 2.7%), *tax-6(p675)* (43.9 ± 1.5%), and *cnb-1(jh103)* (40.9 ± 1.5%) mutant worms compared to that in N2 worms (51.8 ± 0.7%) (Figure 1D).

To confirm the effects of CNA-1/TAX-6 on fat accumulation, we crossed the *Ex[pAK-13](Ptax-6::tax-6::gfp)* strain [27] into the *tax-6(ok2065)* mutant, which is a deletion mutation with more pronounced effects on fat content and growth rate than another two mutations *tax-6(p675)* and *cnb-1(jh103)* (Figure 1D,E). We found that the fat storage indicated by Nile Red staining of fixation and quantified by both the TLC/GC, as well as the size of LD in *tax-6(ok2065);Ex[pAK-13](Ptax-6::tax-6::gfp)* worms, were comparable to that of N2 (Figure 1A–D). In addition to the previous reports [19,20,21], all three calcineurin-deficient mutants displayed retarded development compared to the development of N2 (Figure 1E). Remarkably, the growth rate of *tax-6(ok2065);Ex[pAK-13](Ptax-6::tax-6::gfp)* worms was very similar to that of the N2 worms (Figure 1E). Taken together, these results demonstrate that genetic disruption of calcineurin definitely leads to reduced fat accumulation, which suggests that calcineurin plays a conserved role in regulating of lipid metabolism in *C. elegans*.

### 2.2. Calcineurin Inhibitor Mimics the Effects of Calcineurin Mutations on Fat Accumulation

**Figure 2:**
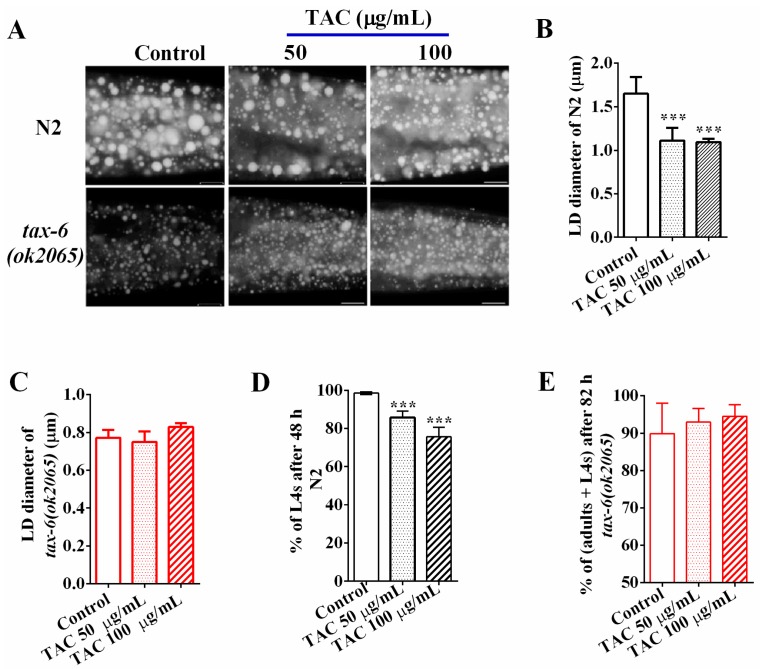
Tacrolimus (TAC) decreases fat accumulation via calcineurin. (**A**) Nile Red staining of fixed worms treated with different concentrations of tacrolimus (TAC). Representative animals are shown with stained lipid droplets (LDs) in the posterior region. In all of the represented animals, the anterior is indicated on the left and the posterior is indicated on the right. Scale bar represents 10 μm; (**B**) The average size of the lipid droplet (LD) in N2 were measured from Nile Red staining of fixed worms from (**A**). *n* = 10 for each worm strain; (**C**) The average size of the lipid droplet (LD) *tax-6(ok2065)* were measured from Nile Red staining of fixed worms from (**A**). *n* = 10 for each worm strain; (**D**) the growth of wild type N2 upon tacrolimus (TAC) treatment or not; and (**E**) The growth of *tax-6(ok2065)* upon tacrolimus (TAC) treatment or not. Data are presented as the means ± SD. * indicates significant difference between worms treated with and without tacrolimus (TAC), *** *p* < 0.001. *n* > 150 worms.

Calcineurin is the target of the immunosuppressant drug tacrolimus (TAC) [11], which has been reported to cause dyslipidemia in clinical applications [7,16]. To examine the effects of tacrolimus (TAC) on fat accumulation in *C. elegans*, we treated wild-type N2 and *tax-6(ok2065*) L1 worms with tacrolimus at concentrations of 0 (control), 50, and 100 μg/mL, and collected worms when they reached adulthood. The Nile Red staining of fixation (Figure 2A) and the quantifications of the average LD size (Figure 2B) showed that the average LD sizes of young adult N2 worms treated with 50 and 100 μg/mL tacrolimus (TAC) were 1.11 ± 0.13 and 1.09 ± 0.04 μm, which were significantly smaller than that of worm treated without tacrolimus (1.65 ± 1.17 μm, 0 μg/mL), indicating that tacrolimus (TAC) displayed the ability to reduce the fat accumulation in N2 worms. However, the reduction effects on fat accumulation and LD size by tacrolimus (TAC) apparently disappeared in *tax-6(ok2065)* mutant worms (Figure 2A,C). Additionally, the percentage of L4 among N2 worms treated with 50 and 100 μg/mL tacrolimus (TAC) for 48 h were 85.69 ± 3.26% and 75.61 ± 4.83%, which were significantly lower than those treated without tacrolimus (TAC) (98.51 ± 1.96%) (Figure 2D). However, there was no difference in growth rate among *tax-6(ok2065)* worms receiving treatments or no treatment of tacrolimus (TAC) (Figure 2E), suggesting that calcineurin is required for the growth inhibition of tacrolimus (TAC). Collectively, these data clearly indicate that pharmacological inhibition of calcineurin via the immunosuppressant drug tacrolimus (TAC), like the genetic disruption of calcineurin, will result in reduced fat accumulation and delayed development in *C. elegans*.

### 2.3. AMPK Antagonizes Calcineurin to Regulate Fat Metabolism

**Figure 3:**
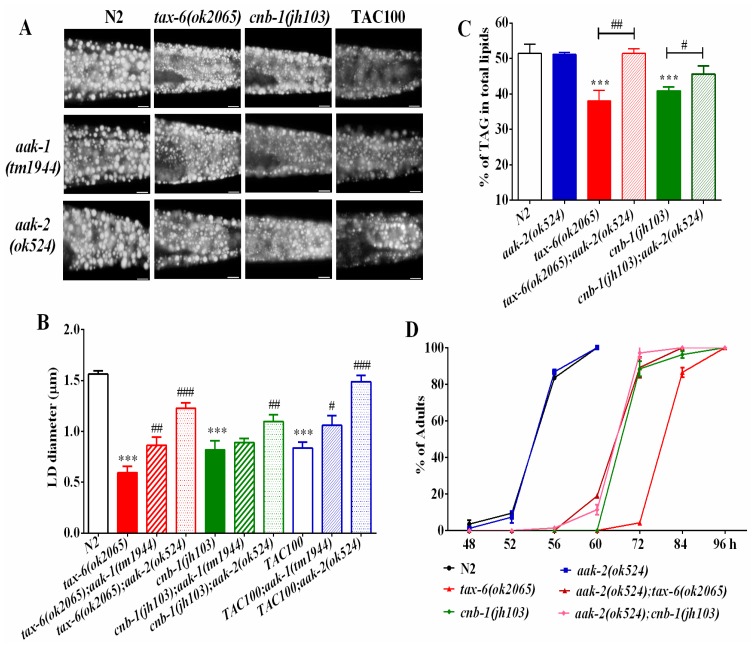
AMPK antagonizes calcineurin to regulate fat metabolism and growth rate. (**A**) Nile Red staining of fixed worms. Representative animals are shown with stained lipid droplets (LDs) in the posterior region. In all of the represented animals, the anterior is indicated on the left and the posterior is indicated on the right. Scale bar represents 10 μm; (**B**) the average size of the lipid droplets (LD) were measured from Nile Red staining of fixed worms from (A). *n* = 10 for each worm strain; and (**C**) the percentage of triacylglycerol (TAG) in total lipids (TAG + phospholipids). Data are presented as the means ± SD of at least three biological repeats; and (**D**) the growth rate of worms. *n* > 200 worms. Data are presented as the means ± SD. * indicates significant difference between wild-type N2 and a specific worm strain, *** *p* < 0.001. ^#^ indicates significant difference between two indicated worm strains, ^#^ *p* < 0.05, ^##^ *p* < 0.01, ^###^ *p* < 0.001.

Phosphatases and kinases generally play opposite actions to fine-tune cellular events. In mammals, calcineurin acts with the AMP-activated kinase (AMPK) antagonistically to regulate energy homeostasis and endoplasmic reticulum stress. Similarly, it also regulates lifespan and adaptive autophagy during oxidative stress [22,28] in an opposite way in *C. elegans*. In *C. elegans*, *aak-1* and *aak-2* encode for the catalytic subunit of AMPK [29,30]. To examine whether the AMPK signaling pathway might antagonize calcineurin in regulating lipid metabolism, we generated *aak-2(ok524);tax-6(ok2065)* and *aak-1(tm1944);tax-6(ok2065)* double mutants. We found that both *aak-2(ok524);tax-6(ok2065)* and *aak-1(tm1944);tax-6(ok2065)* double mutant worms showed increased fat accumulation and lipid droplet size compared to those of *tax-6(ok2065)* single mutant worms (Figure 3A,B), in which *aak-2(ok524)* mutation displayed a better rescue effect than *aak-1(tm1944)* mutation.

Similarly, the lipid droplet size and fat accumulation of *cnb-1(jh103)* mutant worms and tacrolimus (TAC) treated worms also apparently improved in *aak-1* and *aak-2* mutant backgrounds (Figure 3A,B). Consistently, the TAG levels (Figure 3C), as well as the growth rate (Figure 3D) of *aak-2(ok524);tax-6(ok2065)* and *aak-2(ok524);cnb-1(jh103)* double mutant worms, were significantly improved than those of *tax-6(ok2065)* and *cnb-1(jh103)* single mutant worms alone. Taken together, these results suggest that AMPK antagonizes calcineurin to regulate fat accumulation and growth.

### 2.4. Calcineurin Promotes Lipolysis via Adipose Triglyceride Lipase (ATGL-1)

Lipid homeostasis is determined by the balance of lipogenesis and lipolysis. Calcineurin was reported to be involved in insulin/IGF-1 signaling pathway [27], which plays important roles in regulating lipogenesis and fat accumulation in *C. elegans* [31,32]. Calcineurin inhibits the nuclear translocation of forkhead box O (FOXO) transcription factor DAF-16 [33], and the longevity and dauer formation of the *tax-6(ok2065)* mutant was reported to be partially dependent on the DAF-16 activity [27]. However, the Nile Red staining showed that the fat accumulation, lipid droplet size, as well as the growth rate of the *daf-16(mu86);tax-6(ok2065)* double mutants were similar to that of the *tax-6(ok2065)* single mutant (Figure S1A–D), suggesting that the altered fat accumulation and growth is likely to be independent of DAF-16.

**Figure 4:**
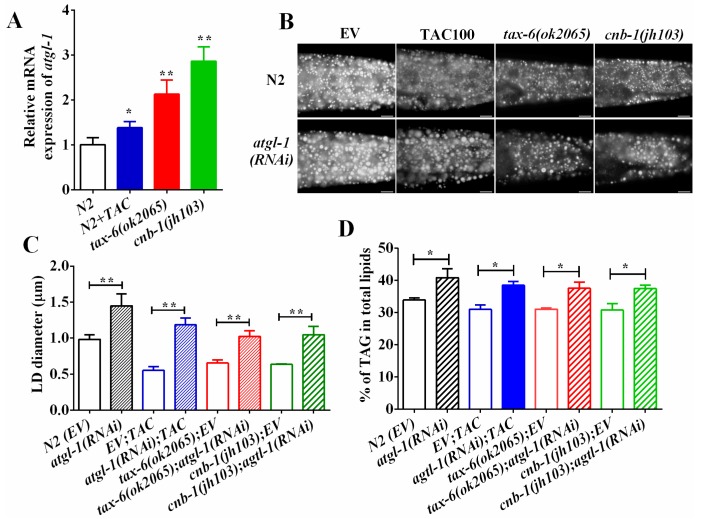
Calcineurin represses ATGL expression and lipolysis. (**A**) The mRNA level of *atgl-1* in wild-type, *tax-6(ok2065)* and tacrolimus (TAC) treated worms; (**B**) Nile Red staining of fixed worms. Representative animals are shown with stained lipid droplets (LDs) in the posterior region. In all of the represented animals, the anterior is indicated on the left and the posterior is indicated on the right. Scale bar represents 10 μm; (**C**) the average size of the lipid droplets (LD) were measured from Nile Red staining of fixed worms from (**A**). *n* = 10 for each worm strain; and (**D**) the percentage of triacylglycerol (TAG) in total lipids (TAG + phospholipids). Data are presented as the means ± SD of at least three biological repeats. * indicates significant difference between two indicated worm strains, * *p* < 0.05, ** *p* < 0.01.

Desnutrin/ATGL hydrolyzes the TAG in the initial rate-limiting step during lipolysis. ATGL has been reported to be a direct target of AMPK [30,34,35]. Since *aak-1* and *aak-2* mutant could restore fat accumulation of calcineurin mutants and inhibitors, we thereby asked whether ATGL was also involved in calcineurin to regulate fat accumulation. We found that the mRNA expression of *atgl-1* was significantly upregulated in *tax-6(ok2065)*, *cnb-1(jh103)* and tacrolimus (TAC) treated worms compared to that of N2 worms (Figure 4A), suggesting that calcineurin inhibits the transcriptional expression of *atgl-1*. Furthermore, RNAi knockdown of *atgl-1* significantly increased the lipid droplet size and the fat accumulation in N2 worms as well as in *tax-6(ok2065)* and *cnb-1(jh103)* mutants and tacrolimus (TAC)-treated worms (Figure 4B–D). Taken together, these lines of results indicate that calcineurin represses ATGL-1 and lipolysis to maintain lipid homeostasis.

## 3. Discussion

Dysfunction of calcineurin in yeast and rodents leads to dyslipidemia. Here, we showed that the genetic disruption of calcineurin consistently reduces the fat accumulation in model organism *C. elegans*. Meanwhile, clinical application of the immunosuppressant drug tacrolimus (TAC), which target calcineurin to inhibit its function [11], causes dyslipidemia [15,16]. Concordantly, we confirmed that treatments of tacrolimus (TAC) also decreases fat accumulation in worms, completely phenocopying the calcineurin mutants. Thus, calcineurin indeed plays an evolutionarily-conserved role in regulating lipid metabolism from yeast, *C. elegans* to mammals.

A well-recognized function of calcineurin is the dephosphorylation of the transcription factor Nuclear Factor of Activated T Cells (NFAT) [36]. Nevertheless, *C. elegans* lacks NFAT homologues [37]. The FOXO transcription factor DAF-16 plays critical roles in regulating lifespan, development and metabolism [38]. Previous work has shown that calcineurin and Ca^2+^/calmodulin-dependent protein kinase II (CAMII) reversely phosphorylated the transcription factor DAF-16 to regulate *C. elegans* lifespan [33]. However, we found that the *daf-16* mutant failed to recover the reduced fat accumulation of the *tax-6(ok2065)* mutant (Figure S1), suggesting that the calcineurin regulation of lipid metabolism is distinct from its DAF-16-dependent lifespan regulation.

Calcineurin was previously reported to antagonize AMPK to regulate lifespan [22], and also suppresses adaptive autophagy during oxidative stress by downregulating the AMPK signaling pathway in *C. elegans* [28]. In this study, we found that the reduced fat accumulation in *tax-6(ok2065)* and *cnb-1(jh103)* mutants, as well as in tacrolimus (TAC) treated worms was significantly increased for the AMPK *aak-2(ok524)* mutant (Figure 3). Therefore, AMPK has the ability to antagonize calcineurin to regulate lipid metabolism, implying the possibility that dysfunction of AMPK may counteract the dyslipidemia effect of tacrolimus (TAC).

The lipase ATGL is a direct target of AMPK [30,34,35]. We found that calcineurin and its inhibitor repress the mRNA expression of *atgl-1*. In mice, disruption ATGL has been shown to increase triglyceride accumulation within multiple tissues [39]. Consistently, RNAi knockdown of *atgl-1* led to fat accumulation in *C. elegans*. Meanwhile, similar to the *aak-2(ok524)* mutant, the RNAi knockdown of *atgl-1* improved the decrease in fat accumulation of calcineurin-defective mutants. In addition, therapeutic concentrations of calcineurin inhibitor tacrolimus (TAC) increase the phosphorylation of hormone-sensitive lipase HSL, which hydrolyzes triglycerides and inhibits lipid storage in human adipose tissue [40]. Therefore, these lines of evidences suggest that calcineurin, as well as its inhibitors, probably regulate lipolysis to modulate lipid metabolism.

In summary, our finding in *C. elegans* revealed a conserved role of calcineurin in the regulation of lipid metabolism, in which calcineurin as well as its inhibitor tacrolimus (TAC) repress lipolysis to maintain lipid homeostasis. Moreover, our works may also provide potential treatment to alleviate tacrolimus (TAC) caused dyslipidemia through antagonizing the AMPK signaling pathway.

## 4. Materials and Methods

### 4.1. Nematode Strains and Growth Conditions

Standard nematode growth media (NGM) was used to maintain *C. elegans* with the *Escherichia coli* OP50 at 20 °C, unless otherwise indicated. All of the strains were cultured and handled using standard methods [41]. N2 Bristol was used as the wild-type strain. The strains used in this study were *tax-6(ok2065)*IV, *tax-6(p675)*IV, *Ex[pAK-13](Ptax-6::tax-6::gfp)* [27,42], *cnb-1(jh103)*V, *aak-2(ok524)*X, *aak-1(tm1944)*III, and *daf-16(mu86)*I. The following strains were made for this study: *aak-1(tm1944)*III;*tax-6(ok2065)*IV, *tax-6(ok2065)*IV;*aak-2(ok524)*X, *aak-1(tm1944)*III;*cnb-1(jh103)*V, *cnb-1(jh103)*V;*aak-2(ok524)*X, and *tax-6(ok2065*IV;*Ex[pAK-13](Ptax-6::tax-6::gfp).*

### 4.2. RNAi Interferance

RNAi interferance assays were performed at 20 °C using the feeding method as previously described [43,44]. Feeding RNAi was performed on NGM plates supplemented with 100 μg/mL ampicillin and 2 mM isopropyl-β-d-thiogalactopyranoside (IPTG) and different RNAi bacteria. Synchronized L1 worms were placed onto above plates with corresponding RNAi bacteria, unless otherwise indicated. *E. coli* HT115 transformed with L4440, an empty RNAi vector, used as a control. The RNAi bacterial strains were obtained from the Ahringer RNAi library(The Wellcome CRC Institute, University of Cambridge, Cambridge, England).

### 4.3. Nile Red Staining of Fixed Nematodes

Young adult nematodes were washed off from the NGM plates, and then fixed and stained with Nile Red as previously described [23,44]. Images were captured using identical settings and exposure time, unless specifically indicated otherwise. At least 15 animals were imaged for each repeat, and three biological repteats were performed.

### 4.4. Lipid Extraction and Analysis

*C. elegans* lipids extraction, separation of triacylglycerol and phospholipids by thin-layer chromatography (TLC), and the analysis of fatty acids by gas chromatography (GC) were performed as previously described [25]. Approximately 100,000 one-day-old young adults were harvested for lipid extration and analysis. Lipids were extracted following the standard procedure. Five milliliters of ice-cold chloroform:methanol (1:1) were added to worm pellets and incubated overnight at −20 °C with occasional shaking. Then, 2.2 mL of Harja’s solution (0.2 M H_3_PO_4_ and 1 M KCl) was added to each sample and shaken vigorously. After centrifugation, the organic phase was removed and dried under nitrogen, then re-suspended in chloroform. Samples were loaded in triplicate on TLC plates, then developed to the top of the plate in the solvent system hexane:diethylether:acetic acid (80:20:2).

Lipids were visualized under UV light after spraying the plate with 0.005% primuline, and spots corresponding to TAG and the major phospholipids were scraped, spiked with a known standard (C15:0), and transesterified for GC analysis to determine the relative levels of TAG and phospholipid fractions. Determination of the fatty acids were run with an Agilent 7890 series gas chromatographer equipped with a 30 × 0.25-mm SP-2380 column (Supelco, Bellefonte, PA, USA), with nitrogen as the carrier gas at 1.4 mL/min, and a flame ionization detector. Four biological replicates were performed for TLC/GC analysis.

### 4.5. Growth Rate

Synchronized L1s were placed onto NGM plates seeded with *E. coli* strain OP50. The number of adults and the total number of worms were scored under a microscope at various time points.

### 4.6. Treatment of Calcineurin Inhibitor

Calcineurin inhibitor tacrolimus (TAC) was purchased from Selleck Chemicals (Houston, TX, USA, Code S5003). Tacrolimus was dissolved in ethanol, and then the solution was poured to the surface of the NGM plates seeded with OP50. Later, synchronized L1 worms were placed and cultured on these plates for further analysis, including visulization of fat accumulation by post-fixed Nile Red staining, determination of fat content by TLC/GC, and growth rate observation, etc.

### 4.7. Quantitative RT-PCR Anaylsis

For RT-qPCR analysis, L4 stage worms were washed with dH_2_O for at least three times to remove the bacteria. dH_2_O was removed as much as possible, and 1 mL RNAiso plus (Takara, Tokyo, Japan, Code 9108) was added and then quickly frozen in liquid nitrogen. RNA extraction was carried out, as previously described [45]. Concentration and purity of RNA samples were determined with a NanoDrop spectrophotometer and gel electrophoresis, respectively. Samples were stored at −80 °C for future use. Reverse transcription was performed with 1 μg RNA per sample using PrimeScript^®^ RT reagent Kit with gDNA Eraser (Takara, Tokyo, Japan, Code RR047A). Diluted cDNA and custom-designed primers were mixed with SYBR Green (TransStart TipTop Green qPCR SuperMix, TransGen Biotech, Beijing, China, Code AQ141), and samples were analysed using a ABI 7900HT analyzer (Applied Biosystems, Foster City, CA, USA). RT-qPCR data presented were from at least three independent biological replicates, and all values were normalized to tubulin as an internal control.

### 4.8. Statistical Analysis

All experiments were conducted at with least three or four biological replicates. Data were analyzed using the software SPSS (SPSS Inc., Chicago, IL, USA), and the results were presented as the mean ± SD. Statistical significance was determined by Student’s *t* test for comparison between the means of two groups, or by a one-way analysis of variance (ANOVA) followed by Tukey’s honest significant difference tests for equal variances and Dunnett’s T3 tests for unequal variances at the α = 0.05 level of significance among multiple groups. Differences were considered as significant when *p* < 0.05.

## Supplementary Materials

Supplementary Materials are available online.
