# Supplementary material for: Calcineurin Antagonizes AMPK to Regulate Lipolysis in Caenorhabditis elegans
Source: Molecules. 2017 Jun 26;22(7):1062. doi: 10.3390/molecules22071062 (PMC6152104; doi:10.3390/molecules22071062)
Supplement: Supplementary file 1 [file molecules-22-01062-s001.pdf]

Supplementary Materials: Calcineurin Antagonizes AMPK to Regulate Lipolysis in *Caenorhabditis elegans*

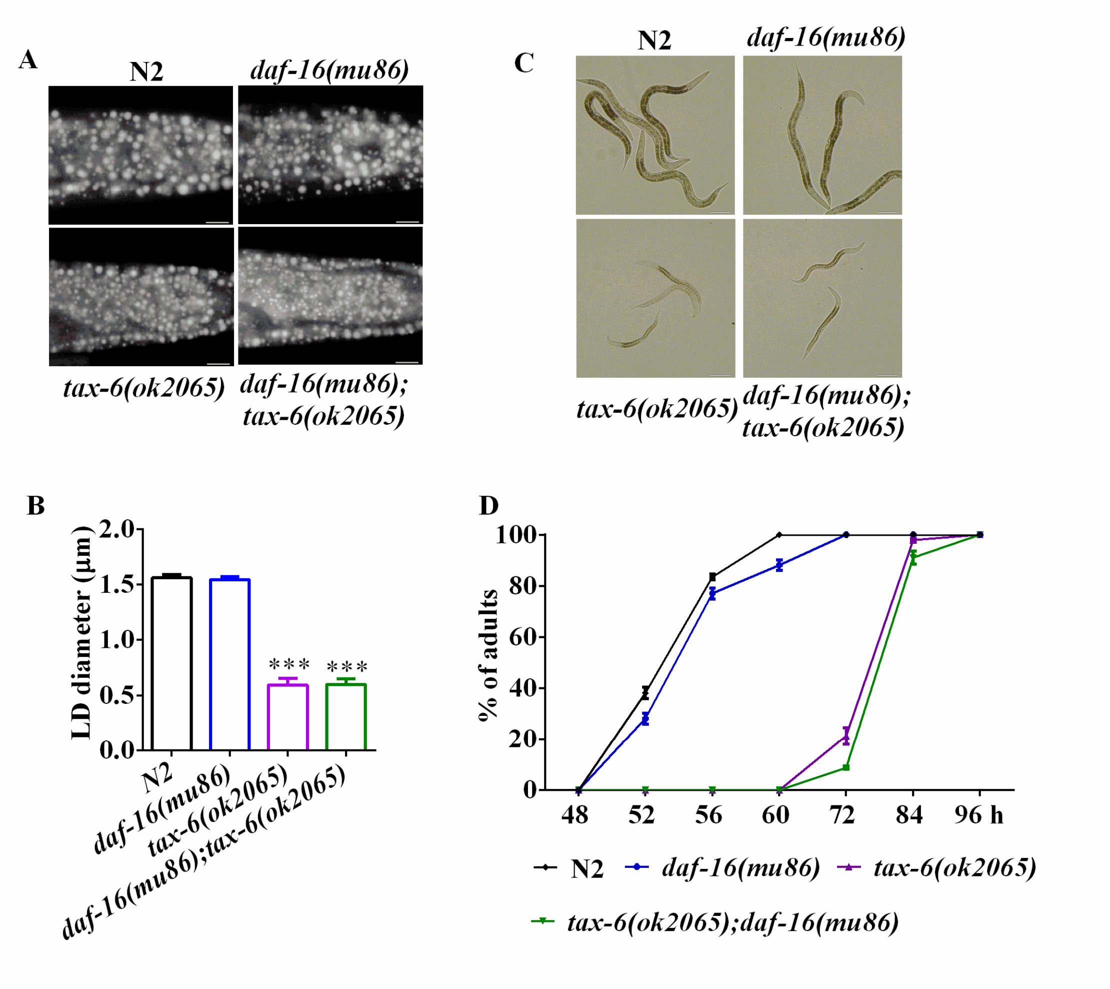

Figure S1. Altered fat accumulation and growth in calcineurin-defective worms may be independent of DAF-16. (**A**) Nile Red staining of fixed worms. Representative animals are shown with stained lipid droplets (LDs) in the posterior region. In all of the represented animals, the anterior is indicated on the left and the posterior is indicated on the right. Scale bar represents 10 μm; (**B**) The average size of the lipid droplets were measured from Nile Red staining of fixed worms from (A); n = 10 for each worm strain; (**C**) The growth of wild-type N2, *tax-6(ok2065)*, *daf-16(mu86)*, and *tax-6(ok2065);daf-16(mu86)* worms; n > 200 worms; and (**D**) The body size of wild-type N2, *tax-6(ok2065)*, *daf-16(mu86)*, and *tax-6(ok2065);daf-16(mu86)* worms after cultivation for 48 hours. Scale bar represents 100 μm. Data are presented as the means ± SD of at least three biological repeats. * indicates significant difference between wild-type N2 and a specific worm strain, *** $p < 0.001$.
